# Hepatic artery stenosis following adult liver transplantation: evaluation of different endovascular treatment approaches

**DOI:** 10.1186/s42155-024-00439-5

**Published:** 2024-04-20

**Authors:** Sagar V. Desai, Balasubramani Natarajan, Vinit Khanna, Paul Brady

**Affiliations:** Department of Interventional Radiology, Jefferson Einstein Hospital, Philadelphia, PA USA

## Abstract

**Purpose:**

To evaluate the efficacy and safety of hepatic artery interventions (HAI) versus extra-hepatic arterial interventions (EHAI) when managing clinically significant hepatic artery stenosis (HAS) after adult orthotopic liver transplantation.

**Materials and methods:**

A single-center retrospective cohort analysis was conducted on liver transplant patients who underwent intervention for clinically significant HAS from September 2012 to September 2021. The HAI treatment arm included hepatic artery angioplasty and/or stent placement while the EHAI treatment arm comprised of non-hepatic visceral artery embolization. Primary outcomes included peri-procedural complications and 1-year liver-related deaths. Secondary outcomes included biliary ischemic events, longitudinal trends in liver enzymes and ultrasound parameters pre-and post-intervention.

**Results:**

The HAI arm included 21 procedures in 18 patients and the EHAI arm included 27 procedures in 22 patients. There were increased 1-year liver-related deaths (10% [2/21] vs 0% [0/27], *p* = 0.10) and complications (29% [6/21] vs 4% [1/27], *p* = 0.015) in the HAI group compared to the EHAI group. Both HAI and EHAI groups exhibited similar improvements in transaminitis including changes of ALT (-72 U/L vs -112.5 U/L, *p* = 0.60) and AST (-58 U/L vs -48 U/L, *p* = 0.56) at 1-month post-procedure. Both treatment arms demonstrated increases in post-procedural peak systolic velocity of the hepatic artery distal to the stenosis, while the HAI group also showed significant improvement in resistive indices following the intervention.

**Conclusion:**

Direct hepatic artery interventions remain the definitive treatment for clinically significant hepatic artery stenosis; however, non-hepatic visceral artery embolization can be considered a safe alternative intervention in cases of unfavorable hepatic anatomy.

**Graphical Abstract:**

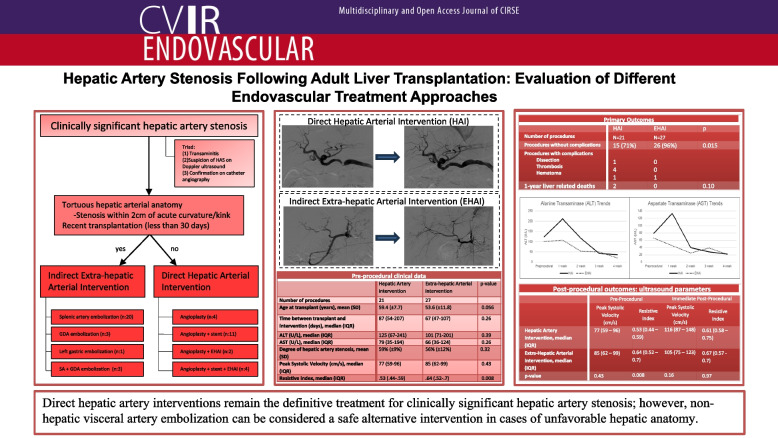

## Introduction

Vascular complications rank among the most common and severe causes of liver graft dysfunction. Among these, hepatic arterial complications involving the anastomosis are the most frequent causes of vascular compromise given the potential for stenosis, thrombosis or pseudoaneurysm formation [1]. This paper focuses on hepatic artery stenosis (HAS), a pathologic entity usually diagnosed within 3–4 months of adult liver transplantation, with a reported incidence ranging from 3.5–9.3% [2–5]. Common causes of HAS include structural (abnormal kinking/angulation, donor-recipient arterial size mismatch), vascular (clamp injury, vaso-vasorum disruption) and immunologic (fibrosis, intimal hyperplasia) complications [6]. Typically, HAS is first detected on noninvasive imaging studies such as spectral Doppler ultrasound (US) or computed tomography angiography (CTA) [7]. Although easily identifiable, the management of HAS is not straightforward. If left untreated, HAS may cause insidious graft dysfunction or graft death owing to the high incidence of thrombosis [6, 8]. Treatment options are broad and multifaceted, including anticoagulation, endovascular intervention, surgical revascularization, and re-transplantation.

The current treatment paradigm leans towards conservative management, with intervention pursued only in clinically significant cases (i.e., transaminitis). Historically, the treatment involved surgical revision with unfavorable outcomes; thus, there has been a recent and sharp turn favoring an endovascular approach. Endovascular interventions include direct hepatic artery angioplasty and/or stent placement, with prior studies demonstrating improved arterial flow on post-procedural ultrasound and significant reductions in elevated liver enzymes [9, 10]. However, direct hepatic artery interventions have high re-intervention rates and may lead to major complications [5, 8–12]. There is increasing literature involving non-hepatic visceral artery embolization (commonly splenic artery embolization) in the post-liver transplant population for indications such as nonocclusive arterial hypoperfusion syndrome (i.e., ‘splenic artery steal’), portal hyperperfusion and refractory ascites [13–18].

However, current literature lacks a comparative study involving both direct hepatic artery interventions (HAI) and indirect extra-hepatic arterial interventions (EHAI) in the setting of HAS. At our institution, management of HAS begins with an inter-disciplinary case-by-case approach involving transplant surgery and interventional radiology. Our working hypothesis is that non-hepatic visceral artery embolization may effectively treat HAS via redistribution of celiac blood flow to mitigate ischemic damage to the liver, as well as reduce the rates of complications associated with HAI. The option to treat symptomatic HAS with EHAI would be particularly helpful in cases of HAS where hepatic arterial anatomy is unfavorable to instrumentation. This study aimed to review the efficacy and safety of indirect and direct hepatic arterial interventions for hepatic artery stenosis to suggest a reasonable treatment algorithm.

## Materials and methods

### Subjects

This single-center, retrospective cohort study included all orthotopic deceased donor liver transplant recipients at our institution, from September 1, 2012, to September 1, 2021, who underwent hepatic artery angiography for suspected HAS presenting with transaminitis. The radiology database (Montage; Nuance, Burlington MA) was searched for visceral angiography reports including “hepatic artery stenosis,” and such reports were cross-referenced for prior liver transplantation. All reports were manually checked for eligibility. Patients who underwent endovascular interventions within 1-year of transplantation with hepatic artery stenosis confirmed on conventional angiography and/or contrast enhanced cross-sectional imaging were included. The exclusion criteria were findings different from, or in addition to, HAS, including hepatic artery thrombosis or concomitant pseudoaneurysm, significant cellular rejection on biopsy, or the presence of an aorto-hepatic jump graft. Patients with imaging characteristics of splenic artery steal phenomenon on US Doppler, such as low or absent diastolic flow in the main hepatic artery, were excluded. Patients who underwent a repeat intervention involving the opposite treatment arm within 1-year of the first intervention (i.e., ‘crossed over’ interventions) were also excluded. This study was approved by the Institutional Review Board (IRB-2021–662 and IRB-2022–939) and was compliant with the standards set forth by the Health Insurance Portability and Accountability Act (HIPAA).

### Data collection

Demographic data included sex, age, time of transplantation, and time of intervention. Pre-procedural clinical data included hemoglobin, creatinine, International Normalized Ratio (INR), alanine transaminase (ALT), and aspartate transaminase (AST). Spectral Doppler ultrasound measurements of peak systolic velocity (PSV) and resistive index (RI) of the hepatic artery at the porta hepatis were collected within a 48-h period immediately prior to and following intervention. The degree of hepatic artery stenosis was quantified, retrospectively, using catheter angiography images to measure luminal diameter at the stenosis compared to a segment of non-affected distal hepatic artery. Major biliary ischemic events and 1-year liver-related survival outcomes were collected. A major biliary ischemic event was counted when intervention for sequelae of biliary tract ischemia, such as percutaneous biliary drainage, was required. Peri-procedural complications were compiled and categorized based on minor and major complications according to the Society of Interventional Radiology Clinical Practice Guidelines [19]. Post-procedural clinical data included the highest ALT and AST values at intervals of 1–7 days, 8–14 days, 15–21 days, and 22–28 days following intervention.

### Definitions and criteria

Clinically significant hepatic artery stenosis requiring intervention was defined as a triad including: (1) transaminitis, (2) suspicion of stenosis on US Doppler and (3) stenosis confirmed with catheter angiography and/or contrast enhanced cross-sectional imaging.

Imaging characteristics on US Doppler suspicious of HAS included decrease in peak systolic velocity, decrease in RI and ‘parvus-tardus’ waveform of the distal hepatic artery, assessed at the porta hepatis. Direct hepatic artery intervention (HAI) was defined as hepatic artery angioplasty with or without stent placement. Indirect extra-hepatic arterial intervention (EHAI) was defined as embolization of a visceral artery originating from the celiac axis, excluding the hepatic artery.

### Procedures

Liver transplant recipients presenting with transaminitis underwent spectral Doppler ultrasound (US) and liver biopsy to determine the etiology of liver injury. Patients without immunological rejection who were suspected to have hepatic artery stenosis underwent conventional angiography to confirm the diagnosis. The decision to intervene directly in the hepatic artery versus embolization of an extra hepatic visceral artery was decided intra-procedurally between the interventional radiologist and the transplant surgeon. The primary factor favoring EHAI was hepatic artery tortuosity such that the stenosis was located within 2 cm of an acute arterial curvature/kink. Additionally, recent transplantation (less than 1 month after surgery) heavily favored EHAI to avoid the risk of hepatic artery anastomotic disruption.

During HAI procedures, the patient was fully anticoagulated with heparin prior to attempted crossing of a HAS, with a goal activated clotting time (ACT) greater than 250 s or double the patient’s baseline. The stenotic segment was crossed with gentle use of a coaxial system including microcatheters 2.4 French or smaller and microwires 0.018 inches or smaller. Verapamil (2.5 mg) was administered intra-arterially via a catheter in cases of hepatic artery spasm. The primary balloon used for angioplasty was the Sterling Monorail (Boston Scientific; Marlborough, MA). Stenting was pursued in cases where angioplasty was insufficient (residual stenosis greater than 20%). The primary stents used included the Rebel bare-metal stent (Boston Scientific; Marlborough, MA) and Viabahn covered stent-graft (Gore; Newark, DE). Following stent placement, the patient was immediately administered 300 mg clopidogrel and continued 75 mg clopidogrel daily for at least 3 months. HAI was considered technically successful when there was less than 20% residual stenosis of the treated hepatic artery, by visual estimate.

For EHAI, proximal splenic embolization was performed just distal to the dorsal pancreatic artery, and embolization of the gastroduodenal artery (GDA) or left gastric artery (LGA) was performed approximately 2 cm distal to the origin. Embolization of the splenic artery was commonly performed with Amplatzer embolization plugs (Abbott Laboratories; Abbott Park, Illinois). Additional embolization of the splenic artery, as well as embolization of the gastroduodenal and left gastric artery, was performed primarily using Concerto detachable embolic coils (Medtronic; Swedesboro, New Jersey). During EHAI, an artery was selected for embolization if it had larger-than-expected vessel caliber compared to the hepatic artery and/or exhibited preferential flow on celiac arteriography. If multiple vessels met this criteria, the largest vessel was first embolized; if this proved insufficient, then the second largest vessel was embolized. No patient had more than two visceral arteries embolized. Successful EHAI was defined by effective redirection of celiac axis flow leading to improved hepatic parenchymal opacification on post-embolization angiography. If EHAI was still insufficient, the decision was made to perform HAI during the same procedure. In such instances of concomitant EHAI and HAI, the ‘combination’ procedure was categorized as a direct intervention and included in the HAI group.

All procedures included within this study were performed by one of three interventional radiologists, each with greater than fifteen years of experience in the field.

### Statistical tests

Baseline pre-procedural data are described as means and standard deviations (SD) or medians with interquartile ranges (IQR). Differences were determined using non-parametric tests for medians and t-tests for means. Complications were described and presented as percentages, with differences determined using the chi-squared test. The median, IQR values, and correlation coefficients with statistical significance were computed for longitudinal clinical and ultrasound data. The analysis was performed using Stata version 17 (Stata; StataCorp, College Station TX). Statistical significance was defined as *p* < 0.05.

## Results

**Fig. 1:**
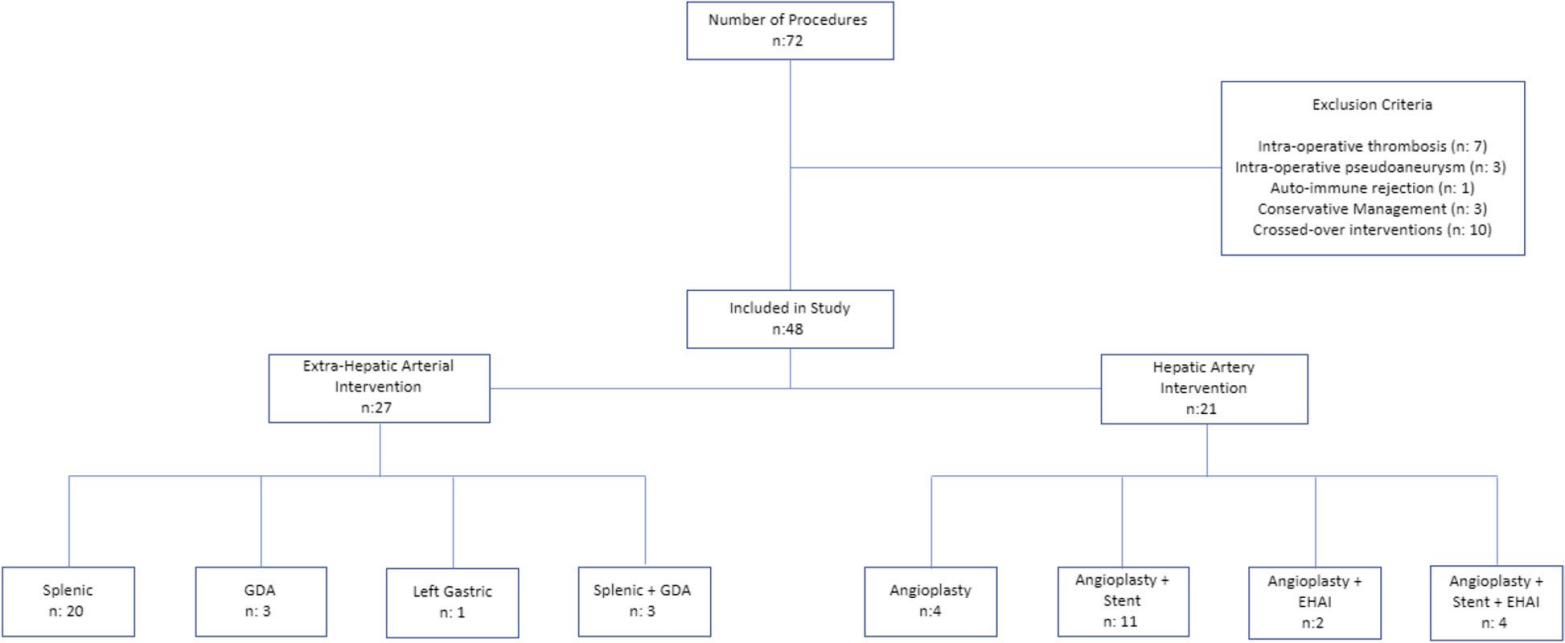
Flowchart of the cohort study design. Procedures performed for suspected symptomatic hepatic artery stenosis during the study period, and subsequent treatments performed when indicated

**Fig. 2:**
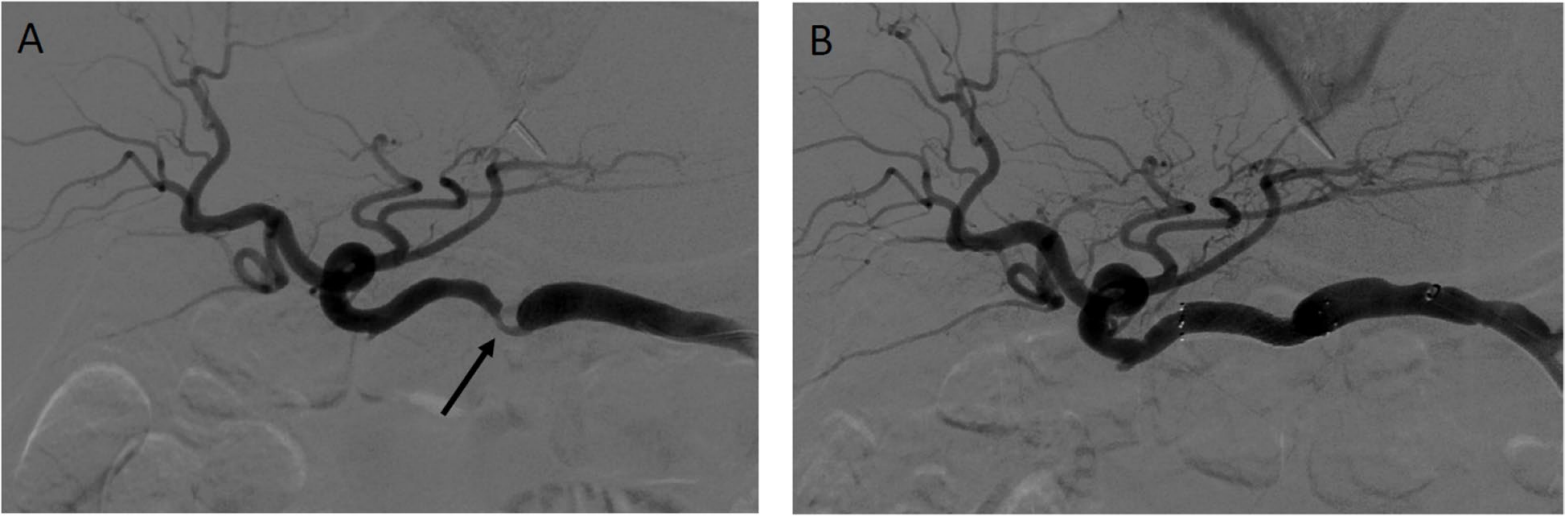
Example of Hepatic Artery. 61-year-old male with liver transplantation (3 months prior) presenting with transaminitis and suspected hepatic artery stenosis. **A** Initial angiography demonstrates that the region of stenosis (arrow) is in a linear segment of hepatic artery which is amenable to direct intervention. **B** Angiography following angioplasty and stenting demonstrates brisks flow through the hepatic artery without evidence of complication

**Fig. 3:**
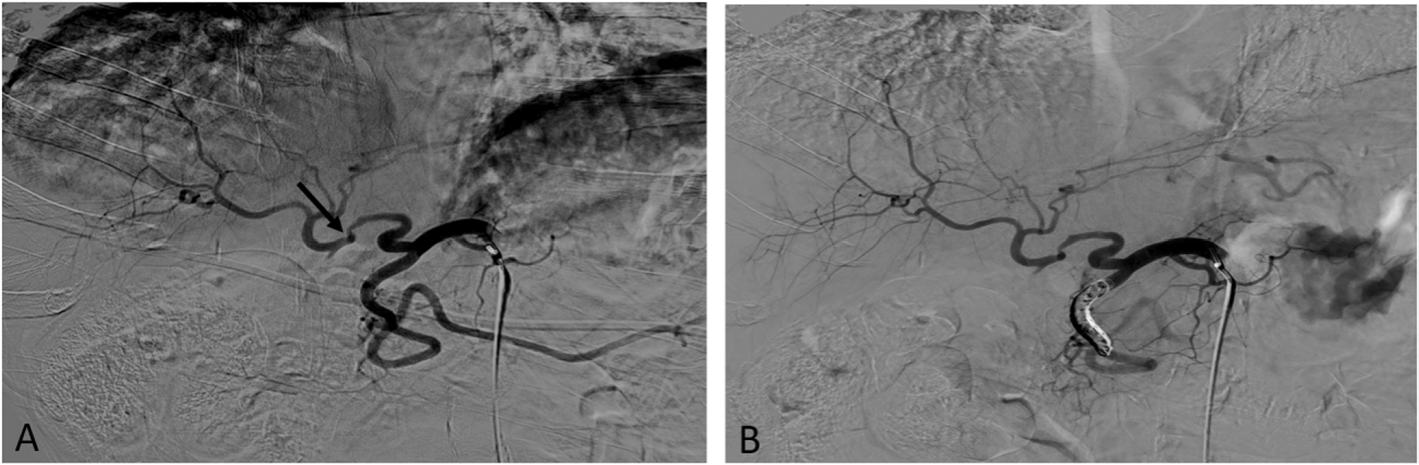
Example of Extra-Hepatic Arterial Intervention. 55-year-old male with history orthotopic liver transplant (2 months prior) presenting with transaminitis in the setting of known hepatic artery stenosis. **A** Common hepatic angiography demonstrating stenosis (arrow), adjacent to region of acute curvature, and dominant gastroduodenal arterial flow. **B** Post-embolization angiography demonstrating improved blood flow through the stenotic hepatic artery and increased hepatic parenchymal blush

**Fig. 4:**
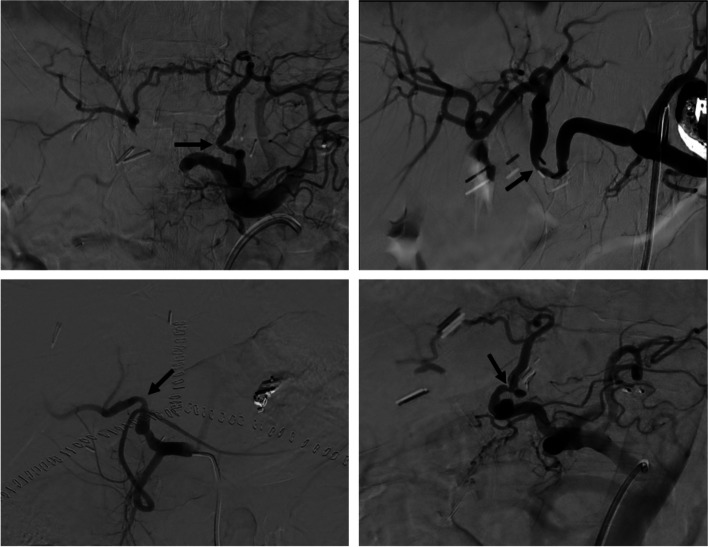
Examples of Anatomy Favoring Extra-Hepatic Arterial Interventions. Four case examples of hepatic artery stenosis (arrows) located adjacent to a region of acute arterial curvature (greater than 90 degrees)

The initial search produced 72 procedures, of which 48 interventions met the inclusion criteria (Fig. 1). Procedures that met the exclusion criteria included intra-procedural findings of thrombosis (7) or pseudoaneurysm (3), auto-immune rejection (1), conservative management (3), or patients who underwent repeat intervention in different treatment arms within the 1-year post-procedural period (10). The HAI treatment arm included 21 procedures in 18 patients and the EHAI treatment arm included 27 procedures in 22 patients. Most EHAI’s involved splenic artery embolization. Out of the 21 HAI’s, six involved angioplasty (of which two had concomitant EHAI) while fifteen involved angioplasty and stenting (of which four had concomitant EHAI). Demographics and pre-procedural data are reported in Table 1. Examples of patients with favorable hepatic artery anatomy treated within the HAI group and tortuous hepatic artery anatomy treated within the EHAI group are shown in Figs. 2 and 3, respectively. Figure 4 demonstrates additional cases of EHAI with difficult hepatic arterial artery in which the stenosis was less than 2 cm from a region acute arterial curvature (less than 90 degrees).

**Table 1:** Pre-procedural clinical data

	Hepatic Artery intervention	Extra-hepatic Arterial intervention	*p*-value
Number of procedures	21	27	
Age at transplant (years), mean (SD)	59.4 (± 7.7)	53.6 (± 11.8)	0.056
Gender (female)	9 (43%)	8 (30%)	0.34
Time between transplant and intervention (days), median (IQR)	87 (54–207)	67 (47–107)	0.26
Procedures requiring repeat intervention	3 (14%)	5 (19%)	0.70
Hb (g/dL), median (IQR)	11.1 (10.3–13.2)	9.4 (8.4–11.2)	0.007
Creatinine (mg/dL), median (IQR)	1 (.9–1.2)	1.1 (.8–1.4)	0.83
ALT (U/L), median (IQR)	125 (67–241)	101 (71–201)	0.39
AST (U/L), median (IQR)	79 (35–194)	66 (36–124)	0.26
INR, median (IQR)	1 (1–1.1)	1.1 (1–1.2)	0.19
Degree of hepatic artery stenosis, mean (SD)	59% (± 9%)	56% (± 12%)	0.32
Peak Systolic Velocity (cm/s), median (IQR)	77 (59–96)	85 (62–99)	0.43
Resistive index, median (IQR)	.53 (.44-.59)	.64 (.52-.7)	0.008

There was no significant difference in most demographic and clinical data between both treatment arms (i.e., gender, age, severity of transaminitis), with exception for pre-procedural hemoglobin. There was a similar degree of hepatic artery stenosis in the HAI group versus the EHAI group (59% [± 9%] versus 56% [± 12], *p* = 0.32). Of note, there was a significantly lower median resistive index in the HAI group than in the EHAI group on pre-procedural evaluation (see Table 1).

One-year liver-related deaths occurred at a non-statistically significant higher rate of 10% (2/21) following hepatic artery interventions versus 0% (0/27) following extra-hepatic arterial interventions (*p* = 0.10). Within the HAI group, liver-related deaths occurred 1 day and 70 days after angioplasty and stent placement. There were two cases of non-liver-related deaths in the EHAI group secondary to sepsis and multiorgan system failure involving coronavirus pneumonia (1) and disseminated candidiasis (1).

There was a significantly higher complication rate of 29% (6/21) in the HAI treatment arm compared to 4% (1/27) in the EHAI treatment arm (*p* = 0.015). The six major post-procedural complications following HAI included intraperitoneal hematoma (1), hepatic artery dissection (1), and thrombosis (4). One case of hepatic artery thrombosis resulted in graft loss requiring re-transplantation within 2 weeks of the procedure. The one major complication following EHAI involved pseudoaneurysm and hematoma development along the hepatic artery after splenic artery embolization. The cause remains unknown; however, it is included as an EHAI complication as it occurred several days following the procedure. Repeat interventions to treat complications were excluded from the analysis because the indication and/or intra-procedural finding was not hepatic artery stenosis. There was one case of major biliary ischemic event in the HAI group. One-year liver-related deaths, ischemic biliary events and post-procedural complications are listed in Table 2. Sub-group analysis was performed in which the six ‘combination’ procedures in the HAI treatment arm were excluded (Table 3). There remained a higher percentage of liver-related deaths (13% [2/15] versus 0% [0/27], *p* = 0.052) and complications (27% [4/15] versus 4% [1/27], *p* = 0.028) in the HAI group versus EHAI group.

**Table 2:** One-year graft survival, biliary ischemic events, and post-procedural complications

	Hepatic Artery Intervention	Extra-Hepatic Arterial Intervention	*p*-value
Number of procedures	*N* = 21	*N* = 27	
Procedures without complications	15 (71%)	26 (96%)	0.015
Procedures with complications			
Dissection	1	0	
Thrombosis	4	0	
Hematoma	1	1	
Biliary ischemic events	1 (5%)	0	
1-year liver related deaths	2 (10%)	0	0.10

**Table 3:** Sub-group analysis of primary outcomes with exclusion of combination procedures

	Hepatic Artery Intervention	Extra-Hepatic Arterial Intervention	*p*-value
Number of procedures	*N* = 15	*N* = 27	
Procedures without complications	11 (73%)	26 (96%)	0.028
Procedures with complications			
Dissection	1	0	
Thrombosis	2	0	
Hematoma	1	1	
Biliary ischemic events	1 (7%)	0	
1-year liver related deaths	2 (10%)	0	0.052

The secondary outcomes regarding spectral Doppler ultrasound parameters of the hepatic artery at the porta hepatis are listed in Table 4. After intervention, a significant improvement in RI values was observed between the HAI group versus the EHAI group (+ 0.11 [IQR + 0.07, + 0.15] vs. + 0.04 [IQR -0.07, + 0.07], *p* < 0.01). Both groups demonstrated nonsignificant increases in median PSV of the hepatic artery following intervention.

**Table 4:** Post-procedural outcomes: ultrasound parameters

	Pre-Procedural		Immediate Post-Procedural	
	Peak Systolic Velocity (cm/s)	Resistive Index	Peak Systolic Velocity (cm/s)	Resistive Index
Hepatic Artery Intervention, median (IQR)	77 (59 – 96)	0.53 (0.44 – 0.59)	116 (87 – 148)	0.61 (0.58 – 0.75)
Extra-Hepatic Arterial Intervention, median (IQR)	85 (62 – 99)	0.64 (0.52 – 0.7)	105 (75 – 123)	0.67 (0.57 -0.7)
*p*-value	0.43	0.008	0.16	0.97

**Fig. 5:**
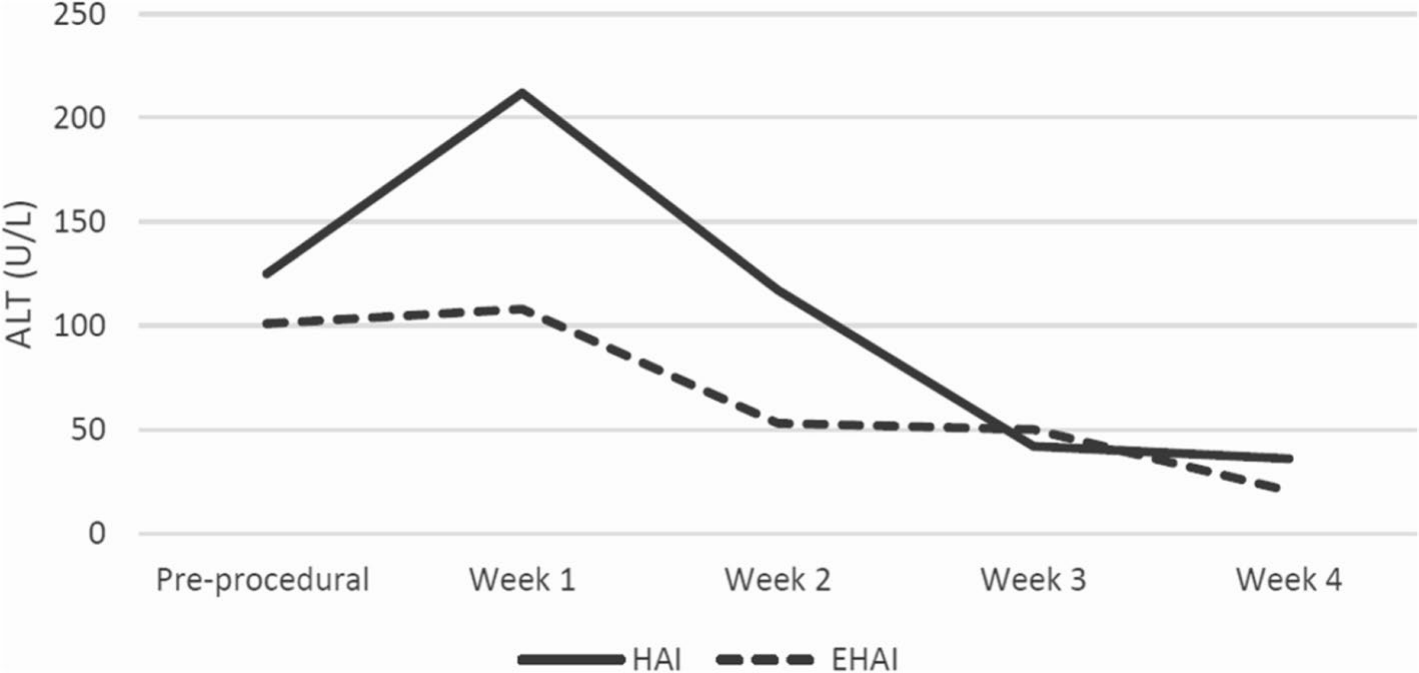
Longitudinal trends of alanine transaminase (ALT). The highest ALT lab values at intervals of 1–7 days, 8–14 days, 15–21 days, and 22–28 days following intervention

**Fig. 6:**
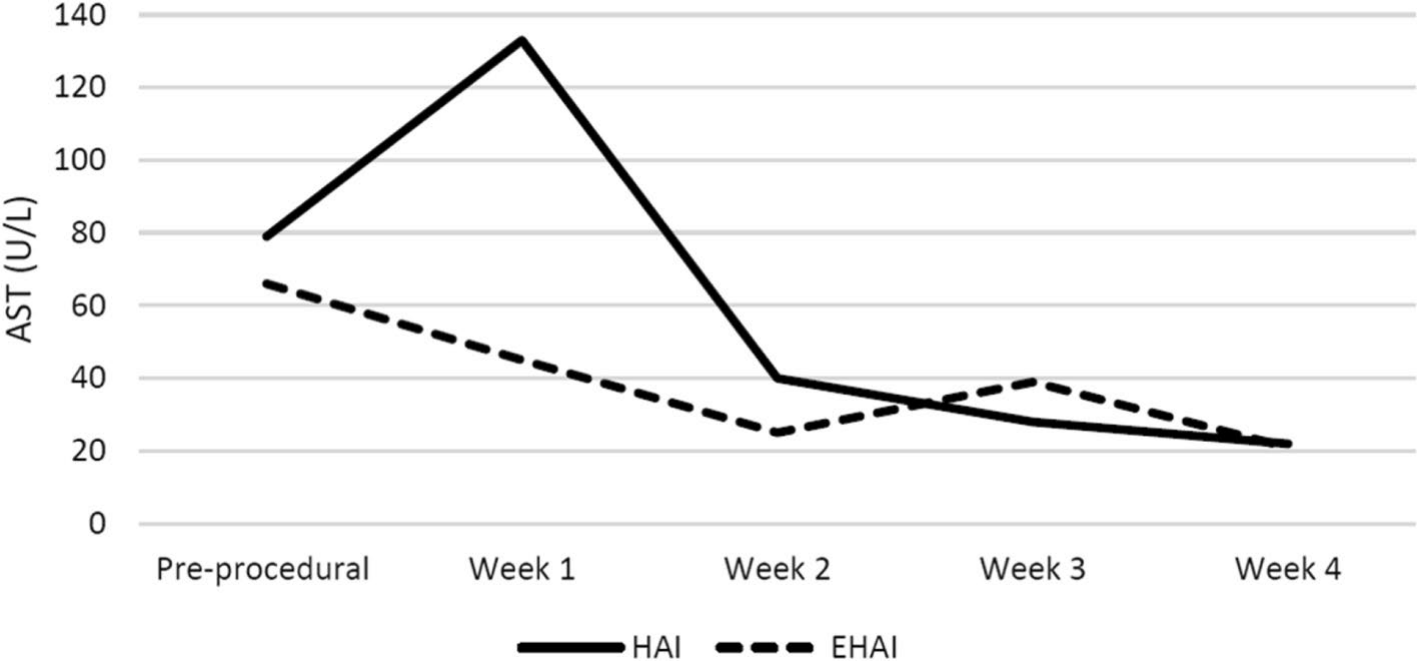
Longitudinal trends of alanine transaminase (AST). The highest AST lab values at intervals of 1–7 days, 8–14 days, 15–21 days, and 22–28 days following intervention

The longitudinal trends in hepatic enzymes are illustrated in Figs. 5 and 6. Pre-procedural transaminitis was not significantly different between the HAI or EHAI groups for both ALT (125 U/L versus 101 U/L, *p* = 0.39) and AST (79 U/L versus 66 U/L, *p* = 0.26). There was an initial spike in liver enzymes one week after intervention in the HAI group; however, trends over the following weeks were similar between both groups. The changes in hepatic enzymes when comparing pre-procedural levels to levels four weeks following intervention were similar between the HAI and the EHAI groups for both ALT (-72 U/L vs -112.5 U/L, *p* = 0.60) and AST (-58 U/L vs -48 U/L, *p* = 0.56) (see Table 5). Of note, the data points for hepatic enzymes included 76% (16/21) of HAI and 85% (23/27) of EHAI procedures at week two and 33% (7/21) of HAI and 30% (8/27) of EHAI procedures at week four.

**Table 5:** Liver enzyme changes in comparison to pre-procedural values

	Hepatic intervention	Extrahepatic intervention	*p*-value
ALT week 1 (U/L), median (IQR)	Δ 8.5 (-14.5, 120)	Δ -6 (-19, 10)	0.12
ALT week 2 (U/L), median (IQR)	Δ -71 (-168.5, -11)	Δ -46 (-113, -19)	0.83
ALT week 3 (U/L), median (IQR)	Δ -57 (-155, -7)	Δ -48 (-126, -22)	0.87
ALT week 4 (U/L), median (IQR)	Δ -72 (-180, -8)	Δ -112.5 (-206.5, -32)	0.60
AST week 1 (U/L), median (IQR)	Δ -.5 (-24, 63.5)	Δ -6 (-21, 12)	0.46
AST week 2 (U/L), median (IQR)	Δ -13.5 (-91.5, -2)	Δ -24 (-48, -14)	0.75
AST week 3 (U/L), median (IQR)	Δ -41 (-102, -12)	D -27 (-61, -2)	0.32
AST week 4 (U/L), median (IQR)	Δ -58 (-182, -40)	Δ -48 (-113, -11)	0.56

The proportion of procedures requiring repeat intervention within the same treatment group was similar between the HAI and EHAI groups (14% [3/21] versus 19% [5/27], *p* = 0.70). When considering that there was a repeat intervention within each of the six ‘combination’ procedures (EHAI immediately followed by HAI), the total proportion of procedures requiring repeat intervention for EHAI grows to 33% (11/33) versus HAI 14% (3/21), *p* = 0.06.

## Discussion

This study compared the safety and efficacy of direct hepatic artery intervention with non-hepatic visceral artery embolization for the treatment of symptomatic hepatic artery stenosis. The treatment modality was primarily determined by the degree of hepatic artery tortuosity such that the stenosis was located less than 2 cm from an acute arterial curvature/kink (see Fig. 4). The results indicate an improved safety profile with EHAI, as demonstrated by decreased liver-related deaths and complications rates when compared to HAI. These conclusions remain consistent when excluding the ‘combination’ procedures from the HAI treatment arm (see Table 3).

Pre-operative hemoglobin levels were statistically different between both treatment arms; however, this was considered an incidental occurrence that did not impact the post-procedural outcomes. Pre-procedural resistive indices measured distal to the stenosis showed statistically significant differences between the groups (see Table 4). However, the other pre-procedural data points used to quantify stenosis, including percentage stenosis, severity of transaminitis, and peak systolic velocity, showed no significant differences between the treatment arms. This homogeneity between patient cohorts in both treatment groups lends support to the notion that celiac blood flow diversion is a valid strategy in the management of HAS.

Previous studies on direct hepatic artery interventions considered progression to hepatic artery thrombosis separate from other procedural complications such as dissection, rupture, or hematoma. When excluding thrombosis, our complication rate of 9.5% (2/21) following hepatic artery angioplasty and/or stenting was similar to previously reported rates of 5.3–12% [5, 8, 10–12]. When comparing the rates of hepatic artery thrombosis following angioplasty and/or stenting for HAS, our rate of 19% (4/21) was slightly lower than previously reported rates of 27–28% [20, 21]. Previous retrospective reviews reported an increase in RI between 0.1 to 0.2 following direct hepatic artery interventions, which is concordant with the + 0.11 change in RI within our HAI treatment group [8, 9].

Regarding the EHAI treatment arm, direct comparisons to existing literature are limited owing to differences in indications for intervention. Previously reported increases in hepatic arterial flow and low complication rates following splenic artery embolization in liver transplant recipients are consistent with our results [15, 16, 18]. To our knowledge, the literature regarding visceral artery embolization to maintain hepatic perfusion in the setting of hepatic artery pathology has been limited to case reports [22].

The present study had some limitations. Due to the retrospective nature of the study, patients received different treatments intraoperatively without prospective randomization. Most patients included in this study had a multi-dimensional treatment approach, including administration of empiric high-dose steroids, prior to a biopsy ruling out rejection. Patients who had undergone transplantation within the past 30-days were preferred candidates of EHAI, potentially creating a systematic bias between the treatment groups. However, the difference in time between transplant and intervention for both groups does not show statistical significance. Improvements in equipment over the course of this 9-year study period have influenced treatment decisions and technical feasibility. For example, Amplatzer embolization plugs (Abbot Laboratories; Abbott Park, Illinois) often used in EHAI have improved from 12 French catheters delivery systems at the beginning of this study to 5 French catheter delivery systems.

Post-transplant hepatic arterial stenosis is a challenging problem with limited published data to guide decision making. Hepatic artery angioplasty and/or stenting is the established endovascular treatment as it directly addresses the pathology; however, instrumentation of the hepatic artery remains a dangerous maneuver with potentially fatal consequences. Therefore, adjunctive and alternative treatment options should be explored and employed, whenever possible. Current indications for non-hepatic visceral artery embolization in post-liver transplant patients are limited to hemorrhage, steal syndrome, and portal hyperperfusion without concomitant hepatic arterial pathology. Based on the results of this study, EHAI demonstrates efficacy in the treatment of symptomatic HAS by using a safe, relatively common, embolization technique. Therefore, the authors of this study suggest that the indications for non-hepatic visceral artery embolization should be expanded to include symptomatic HAS.

## Conclusion

In summary, 22 of the 40 patients with clinically significant HAS were successfully managed with EHAI and were able to avoid direct hepatic artery intervention. Therefore, we suggest that in cases of difficult hepatic arterial anatomy, extra-hepatic visceral artery embolization can be considered as an alternative intervention before attempting instrumentation of the hepatic artery. Further research involving a multi-center registry with long-term results would be helpful in establishing this alternative treatment approach for hepatic artery stenosis following adult liver transplantation.

## Data Availability

The datasets used and/or analyzed during the current study are available from the corresponding author on reasonable request.

## References

[1] Piardi T, Lhuaire M, Bruno O (2016). Vascular complications following liver transplantation: a literature review of advances in 2015. World J Hepatol.

[2] Abbasoglu O, Levy MF, Vodapally MS (1997). Hepatic artery stenosis after liver transplantation-incidence, presentation, treatment, and long term outcome1. Transplantation.

[3] da Silva RF, Raphe R, Felício HC (2008). Prevalence, treatment, and outcomes of the hepatic artery stenosis after liver transplantation. Transpl Proc.

[4] Frongillo F, Grossi U, Lirosi MC (2013). Incidence, management, and results of hepatic artery stenosis after liver transplantation in the era of donor to recipient match. Transpl Proc.

[5] Goldsmith LE, Wiebke K, Seal J (2017). Complications after endovascular treatment of hepatic artery stenosis after liver transplantation. J Vasc Surg.

[6] Chen J, Weinstein J, Black S (2014). Surgical and endovascular treatment of hepatic arterial complications following liver transplant. Clin Transplant.

[7] Frongillo F, Lirosi MC, Nure E (2015). Diagnosis and management of hepatic artery complications after liver transplantation. Transpl Proc.

[8] Saad WEA, Davies MG, Sahler L (2005). Hepatic artery stenosis in liver transplant recipients: Primary treatment with percutaneous transluminal angioplasty. J Vasc Interv Radiol.

[9] Hamby BA, Ramirez DE, Loss GE (2013). Endovascular treatment of hepatic artery stenosis after liver transplantation. J Vasc Surg.

[10] Jarmila L, Jan P (2010). Percutaneous transluminal angioplasty of hepatic artery stenosis in patients after Orthotopic Liver Transplantation: Mid-term results. Cardiovasc Intervent Radiol.

[11] Kodama Y, Sakuhara Y, Abo D (2006). Percutaneous transluminal angioplasty for hepatic artery stenosis after living donor liver transplantation. Liver Transpl.

[12] Sabri SS, Saad WE, Schmitt TM (2011). Endovascular therapy for hepatic artery stenosis and thrombosis following liver transplantation. Vascu Endovasc Surg.

[13] Nüssler N, Settmacher U, Neuhaus P (2003). Diagnosis and treatment of arterial steal syndromes in liver transplant recipients. Liver Transpl.

[14] Vogl T, Pegios W, Balzer J, Lobo M, Neuhaus P (2001). Arterial steal syndrome in patients after liver transplantation: transarterial embolization of the splenic and gastroduodenal arteries. Rofo.

[15] Uflacker R, Selby JB, Chavin K, Rogers J, Baliga P (2002). Transcatheter splenic artery occlusion for treatment of splenic artery steal syndrome after orthotopic liver transplantation. Cardiovasc Intervent Radiol.

[16] Presser N, Quintini C, Tom C (2015). Safety and efficacy of splenic artery embolization for portal hyperperfusion in liver transplant recipients: a 5-Year experience. Liver Transpl.

[17] Eipel C, Abshagen K, Vollmar B (2010). Regulation of hepatic blood flow: the hepatic arterial buffer response revisited. World J Gastroenterol.

[18] Quintini C, D'Amico G, Brown C (2011). Splenic artery embolization for the treatment of refractory ascites after liver transplantation. Liver Transpl.

[19] Khalilzadeh, O., Baerlocher, M. O., Shyn, P. B., et al. (2017). Proposal of a new adverse event classification by the Society of Interventional Radiology Standards of Practice Committee. J Vasc Intervent Radiol. 28(10). 10.1016/j.jvir.2017.06.019 10.1016/j.jvir.2017.06.019 28757285

[20] Naidu S, Alzubaidi S, Knuttinen G (2021). Treatment of hepatic artery stenosis in liver transplant patients using drug-eluting versus bare-metal stents. J Clin Med.

[21] Rajakannu M, Awad S, Ciacio O (2016). Intention-to-treat analysis of percutaneous endovascular treatment of hepatic artery stenosis after orthotopic liver transplantation. Liver Transpl.

[22] Ricci K, Asharf E-H (2018). The use of splenic artery embolization to maintain adequate hepatic arterial inflow after hepatic artery thrombosis in a split liver transplant recipient. Int J Surg Case Rep.

